# CSF neurofilament light chain concentration in patients with delirium following hip fracture: a multicenter prospective study

**DOI:** 10.1186/s12877-026-07630-4

**Published:** 2026-05-19

**Authors:** Irit Titlestad, Leiv Otto Watne, Kaj Blennow, Henrik Zetterberg, Marius Myrstad, Emilija Dunjic, Christian Thomas Pollmann, Adi Karabeg, Lene B. Solberg, Ane-Victoria Idland, Allan Gulestøl, Aasmund Godø, Bjørn Erik Neerland, Lasse M. Giil, Nathalie Bodd Halaas

**Affiliations:** 1 Department of Clinical Medicine, University of Bergen, Bergen, Norway; 2 Neuro-SysMed, Department of Internal Medicine, Haraldsplass Deaconess Hospital Bergen, Ulriksdal 8, Bergen, 5009 Norway; 3 Oslo Delirium Research Group, Institute of Clinical Medicine, University of Oslo, Oslo, Norway; 4 Department of Geriatric Medicine, Akershus University Hospital, Lørenskog, Norway; 5 Department of Psychiatry and Neurochemistry, Institute of Neuroscience and Physiology, The Sahlgrenska Academy, University of Gothenburg, Gothenburg, Sweden; 6 Clinical Neurochemistry Laboratory, Sahlgrenska University Hospital, Mölndal, Sweden; 7 Pitié-Salpêtrière Hospital, Paris Brain Institute, ICM, Sorbonne University, Paris, France; 8 Neurodegenerative Disorder Research Center, Division of Life Sciences and Medicine, Department of Neurology, Institute On Aging and Brain Disorders, University of Science and Technology of China and First Affiliated Hospital of USTC, Hefei, People’s Republic of China; 9 Department of Neurodegenerative Disease, UCL Institute of Neurology, Queen Square, London, UK; 10 UK Dementia Research Institute at UCL, London, UK; 11 Hong Kong Center for Neurodegenerative Diseases, InnoHK, Hong Kong China; 12 Wisconsin Alzheimer’s Disease Research Center, School of Medicine and Public Health, University of Wisconsin, University of Wisconsin-Madison, Madison, WI USA; 13 Department of Internal Medicine, Baerum Hospital Vestre Viken Hospital Trust, Gjettum, Norway; 14 Department of Orthopaedic Surgery, Baerum Hospital, Vestre Viken Hospital Trust, Gjettum, Norway; 15 Department of Anesthesiology, Baerum Hospital, Baerum, Norway; 16 Department of Orthopaedic Surgery, Akershus University Hospital, Lørenskog, Norway; 17 Department of Orthopedic Surgery, Akershus University Hospital, Kongsvinger, Norway; 18 Division of Orthopaedic Surgery, Oslo University Hospital, Oslo, Norway; 19 Department of Anesthesiology, Akershus University Hospital, Lørenskog, Norway; 20 Department of Anaesthesiology, Oslo University Hospital, Oslo, Norway; 21 Department of Anaesthesiology, Diakonhjemmet Hospital, Oslo, Norway; 22 Department of Clinical Science, University of Bergen, Bergen, Norway; 23 Department of Geriatric Medicine, Oslo University Hospital, Ullevål hospital, Building 20, 4th floor, Nydalen, PB 4950 Norway

**Keywords:** Delirium, Mortality, Neurofilament light, Cerebrospinal fluid

## Abstract

**Background:**

Studies suggest delirium is associated with neuronal injury, which may further raise mortality risk. Neuronal injury can be assessed by measuring neurofilament light chain (NFL) concentrations in cerebrospinal fluid (CSF). This study aimed to investigate the association of CSF-NfL with delirium and mortality in patients with hip fracture.

**Methods:**

The study comprised two prospective cohorts of 548 hip fracture patients with per-operative CSF samples. CSF-NfL concentrations were measured using a commercial ELISA. Delirium was assessed daily from admission until the fifth postoperative day and survival censored at one year. The multivariable analyses (Logistic and Cox regression) were adjusted for age, sex, glomerular filtration rate, dementia status, comorbidity, and Activities of Daily Living. Additionally, Cox regression was adjusted for delirium.

**Results:**

In total, 259 (52%) patients developed delirium. In univariate analysis, CSF-NfL was higher among patients with delirium (2116 pg/ml versus 1366 pg/ml, odds ratio (OR) 2.21, (95% confidence interval (CI) [1.75,2,78], *P* < 0.001). In adjusted analysis, CSF-NfL was not significant (OR 1.29, [0.92,1.81], *P* = 0.128) and only remained significantly associated with delirium in the subgroup of patients without dementia (OR 1.84, [1.17, 2.89], *P* = 0.007). In unadjusted analysis of mortality, CSF-NfL was significantly associated with death at one year (hazard ratio (HR) 1.60, [1.37, 1.87], *P* < 0.001) but not in adjusted analysis (HR 1.03 [0.84, 1.26], *P* = 0.736).

**Conclusions:**

Our findings show that CSF-NfL concentrations were associated with delirium in patients without pre-existing dementia, suggesting possible undiagnosed dementia or, less likely, delirium-related neuronal injury. The CSF-NfL-associated mortality hazard was non-significant after adjustment, mainly for delirium. Thus, the clinical context must be considered when studying CSF-NfL and delirium.

**Supplementary Information:**

The online version contains supplementary material available at 10.1186/s12877-026-07630-4.

## Introduction

Delirium is an acute neuropsychiatric syndrome characterized by fluctuating impairments in cognition with altered attention and awareness, triggered by illness and/or injury [1, 2]. This condition is associated with longer hospital stays [3–5], increased risk of dementia [6, 7], and exacerbation of existing dementia [1, 7, 8]. Indeed, a recent meta-analysis reported three-fold greater odds for mortality in patients with delirium [9].

The pathophysiological processes underpinning delirium are still poorly understood, putting major constraints on the development of preventive and treatment measures [1]. However, increasing efforts have been made recently to understand the molecular pathways of delirium by studying cerebrospinal fluid (CSF) biomarkers, and notably neurofilament light chain (NfL) [10].

NfL is a cytoskeletal protein exclusively found in neurons [11]. The release of NfL into extracellular fluids in the CNS occurs from the neuron axon into the CSF and subsequently into the peripheral blood [12]. Increased concentrations of NfL have been measured in CSF and blood in humans [11, 13, 14] and in animal models [15] following neuroaxonal damage. Indeed, elevated NfL concentrations in CSF and/or blood have been observed in a wide range of neurological conditions, including Alzheimer’s disease and other neurodegenerative disorders [13].

Previous studies investigating the association between delirium and NfL are limited to blood NfL measurements, except for two studies that included CSF samples [16, 17]. CSF samples are likely to reflect processes in the brain more accurately and are thus of considerable interest. In blood, elevated NfL concentration has been found in delirium by Halaas et al. [16] (*N* = 314 hip fracture patients, 49% with dementia), Saller et al. [18] (*N* = 9 non-demented cardiac surgery patients), Casey et al. [19] (*N* = 108 older surgical patients), Ballweg et al. [20], (*N* = 114 older adults surgical patients), Page et al. [21] (*N* = 142 ICU patients, 13% with dementia), Krogseth et al. [22] (*N* = 186, population based-cohort) and Fong et al. [23] (*N* = 108 non-demented older adults undergoing major surgery). However, there is a notable lack of studies specifically examining the relationship between NfL concentrations and delirium according to dementia status. Notably, Halaas et al. [16] showed that patients with dementia had significantly higher serum NfL levels in the presence of delirium, compared to those without dementia. This represents an important knowledge gap that warrants further investigation.

A previous study by Halaas et al. [16] included some of the participants from the current study (cohort 1, *N* = 130). The authors reported that unlike in blood, the differences in CSF-NfL concentration between hip fracture patients with and without delirium did not reach significance [16]. However, CSF-NfL tended to be higher among patients with delirium and correlated positively with blood NfL [16]. Additionally, the study by Tsui et al. [17] included 35 geriatric medicine inpatients, of whom 27 had dementia, and found an association between elevated CSF-NfL concentration and persistent delirium.

Several studies have identified that elevated CSF-NfL [24–27] and blood NfL are associated with mortality in both individuals with and without brain disease [21, 28, 29], including those diagnosed with dementia [13]. However, the association between CSF-NfL concentration and mortality has mainly been investigated among individuals with chronic neurodegenerative diseases and not acute illness [24–27]. Studies regarding the relationship between mortality and NfL among patients with delirium are limited.

Studies show that NfL concentrations may also be affected by factors outside the nervous system including inflammation, blood loss, diabetes, age, sex and renal function [14, 16, 19, 30–32]. Importantly, age, inflammation and cognitive impairment are also linked to delirium [1, 4] and mortality [1, 6, 7, 9, 33]. Some studies suggest that the positive association between age and NfL occurs due to neuronal loss during brain ageing [30].

We aimed to investigate four main objectives: First, to examine the association between CSF-NfL concentration and delirium in patients with acute hip fracture. Second, to assess if there is an association between CSF-NfL concentration and one-year mortality. Third, to investigate associations of CSF-NfL with delirium and mortality according to dementia status. Fourth, to assess whether known clinical factors associated with NfL and delirium explain the observed associations between CSF-NfL concentration, delirium and mortality.

## Materials and methods

### Study participants

The study included data from 548 participants from two prospective cohort studies of surgical patients with acute hip fracture with available CSF samples. The first study was conducted between 2009 and 2012 and included 131 patients from one hospital in Oslo, Norway [34]. The associations between delirium and NfL in serum and CSF in the first cohort were previously published [16]. The second study was conducted between 2016 and 2020 and included 417 patients from four hospitals in the Oslo region, Norway. All patients who were acutely admitted to the participating hospitals due to hip fracture, and underwent surgery in spinal anesthesia, were eligible for inclusion. In the first cohort, patients with a hip fracture resulting from a high-energy trauma (defined as a fall from higher than one meter) or patients considered too frail to survive the surgery were excluded from the study [35]. The differences in exclusion criteria between the cohorts arise from differences in the type of the studies. The first cohort was a randomized controlled trial evaluating the effect of treatment on delirium prevention. The main intervention comprised Comprehensive Geriatric Assessment, involving daily interdisciplinary team meetings for treatment coordination and discharge planning. However, the intervention did not affect the occurrence of delirium [35]. The second cohort was part of an observational study that did not involve any intervention, allowing all patients who underwent surgery under spinal anesthesia to be eligible for inclusion.

Patients with subsyndromal delirium (*n* = 51, see below for definition) were excluded from the main analysis of delirium in the current study, as these cannot be classified as delirium cases or controls (non-delirium cases) in biomarker studies [36]. Subsyndromal delirium cases were only included in the survival analysis and classified as non-delirium cases. Mortality was registered using the all-cause mortality from the Norwegian Cause of Death Registry. The patients were censored at one-year following admission for hip fracture.

### Data collection

#### Delirium screening

All patients were screened for delirium preoperatively and daily until the fifth postoperative day, as described in more detail previously [37, 38]. Patients who did not experience delirium during this period were not routinely screened for it afterwards. Briefly, delirium was assessed once a day (mainly on weekdays). In the first cohort, trained nurses or physicians assessed delirium by using the Confusion Assessment Method (CAM) [39]. The total CAM score was determined through a 10 to 30-minute interview with the patients, along with supplementary information from nurses and the patients’ relatives. To identify possible delirium episodes, two of study physicians (LOW and BEN) independently reviewed all relevant medical record documentation. For the second cohort, delirium was evaluated using the Diagnostic and Statistical Manual of Mental Disorders 5 (DSM-5) [2]. Trained nurses conducted this evaluation. The same study physicians from the first cohort (LOW and BEN) independently verified that all DSM-5 criteria were met.

Delirium cases were categorized as preoperative delirium (prevalent delirium), incident delirium (delirium that developed postoperatively during hospitalization), and subsyndromal delirium. Subsyndromal delirium refers to a condition where patients experience several symptoms of delirium but do not meet all the diagnostic criteria for delirium [16, 38].

#### Clinical data

The Informant Questionnaire on Cognitive Decline in the Elderly (IQCODE) [40] was used to assess pre-fracture dementia status, with a cutoff of ≥ 3.44 indicating dementia [41]. The IQCODE is a validated dementia assessment tool frequently used in clinical studies with hospitalized patients and has a sensitivity of about 0.80 [40]. In cases with missing IQCODE assessments (*n* = 38), the research team used information from the hospital medical records to establish dementia status, including previous diagnoses, reports from outpatient clinics and municipal care. Furthermore, the pre-fracture Activities of Daily Living (ADL) was assessed by using the Barthel ADL Index [42]. The ADL score ranges from 0 to 20, with lower scores indicating less physical disability [43]. Furthermore, we used the preoperative American Society of Anesthesiologists (ASA) physical status classification [44] as a proxy measure of comorbidity, with ASA score III-IV indicating high morbidity [45, 46]. The ASA classification system is considered to have high validity and may contribute to predicting postoperative mortality [47].

#### Sampling and biochemical analyses of cerebrospinal fluid

CSF samples were collected preoperatively, as described in more detail previously by Watne et al. [48]. Briefly, CSF samples were collected at the onset of spinal anesthesia before administering the anesthetic agents. The samples were centrifuged, and the supernatants were aliquoted and stored in polypropylene tubes at -80° C. All samples were shipped on dry ice for analysis at the Clinical Neurochemistry Laboratory at Sahlgrenska University Hospital. CSF-NfL concentration was measured using a commercial ELISA (UmanDiagnostics, Umeå, Sweden) for cohort 2 and an older assay for cohort 1 [49].

Technicians were blinded to clinical data. In Cohort 1, 11 samples were measured with both assays with Lin’s concordance correlation of absolute agreement of 0.94 [50]. The median [interquartile range (IQR)] CSF-NfL concentrations were not significantly different between the cohorts (Cohort 1, *n* = 131, median 1804 [1144, 2883] versus cohort 2 (*n* = 417) median 1734 [1156, 2816], *P* = 0.534, Mann-Whitney U test).

#### Statistics

The main analysis grouped cases of prevalent and incident delirium together, excluding those with subsyndromal delirium. The CSF-NfL concentrations had a right (positively) skewed distributions, violating the assumption of normality made by most parametric analyses. Thus, a univariate association between CSF-NfL and delirium was determined using the non-parametric Mann-Whitney U test. ADL function was similarly skewed and thus assessed using the Mann-Whitney U test (any missing cases were imputed as the mean after transformation, see further below). The association between delirium and normally distributed variables, including, age and glomerular filtration rate (eGFR) were assessed using the student’s T-test. Further, the association between delirium and categorical variables, including sex and IQCODE (cutoff was used) was assessed using the Pearson’s *X*^2^.

For the univariate analyses, we aimed to estimate an effect size that would be comparable for normally and non-normally distributed variables and categories. To achieve this, we normalized the area under the curve (AUC) obtained from the Mann-Whitney U test or logistic regression so that zero represented no effect. The resulting Gini coefficient (GC) was calculated as ((AUC×2)–1) [51]. The subsequent scale (0 to 1 or 0 to -1 (i.e., positive or negative association)) represents effect sizes that can be considered as weak (≥ 0.1 to < 0.3), moderate (≥ 0.3 to < 0.4) and strong (≥ 0.4) [52].

Prior to multivariate analyses, CSF-NfL was log-transformed as the log-normal distribution gave the best fit after examining Tuckey’s ladder of powers [53]. Due to differences in assay versions used for the measurement of NfL, although these were closely correlated in the subsample where both were available, we adjusted all analyses for cohort as a covariate.

The previous study by Halaas et al. [16], which utilized data from the first cohort should be considered explorative in nature as only 130 participants had measured CSF-NfL. Using estimates of obtained power to detect an odds ratio (OR) of 1.5, a moderate effect size, an α of 0.05 for a two-tailed test at this sample size using logistic regression and a normally distributed biomarker, the obtained power in Halaas et al.^16^ was estimated at 0.59. In contrast, the current study, using the same set-up for power analysis for logistic regression, achieved a statistical power of 0.97 (*N* = 497) and 0.82 (*n* = 275) for the subsample without dementia.

Next, we performed multivariable analyses using logistic regression with delirium as the outcome. Potential confounders were considered a priori to be key clinical factors of delirium that could be associated with CSF-NfL and included age, sex, eGFR, dementia (IQCODE ≥ 3.44), ASA score (III-IV versus I-II) and ADL function (ADL is associated with NfL in patients without dementia [54]). Multicollinearity was checked. Following the main analysis, we stratified the analysis based on the presence of dementia. We estimated the most influential confounders by estimating the per-covariate percentage change in estimate where we considered > 10% as influential. The change in estimate was calculated as (^crude^OR - ^adjusted^OR)/^crude^OR. Further, we aimed to identify the most important confounders using a multivariate backward selection approach based on the change-in-estimate, as implemented in the Stata package “Epiconf” [55, 56].

We used the exact same approach using Cox regression analysis to estimate the association between NfL, delirium, confounders and one-year mortality. Subgroup analysis was considered for patients with and without evidence of dementia. The analysis was performed by introducing interactions between CSF-NfL and dementia (IQCODE > 3.44) using logistic regression with delirium as the outcome or Cox regression for mortality. Delirium, dementia, age and sex had no missing data. For eGFR, 14 cases (2.6%) were imputed as the sex-specific mean (mean imputation), and 34 cases missing (6.2%) ADL as the overall median, to preserve overall sample size in multivariate analyses. Given the low percentage of missing data, imputation of the central tendency is unlikely to significantly affect estimates [57]. We used simple imputation procedures as the proportion of missing data was within commonly accepted thresholds (5–10%) [58]. ADL was registered by interviewing informants and thus missing data meant the study could not reach informants, which we considered a missing-completely at random (MCAR) mechanism, that is associated with less bias using simple imputation methods. A complete case analysis was also conducted, yielding comparable results and the missing data were for covariates only.

**Fig. 1:**
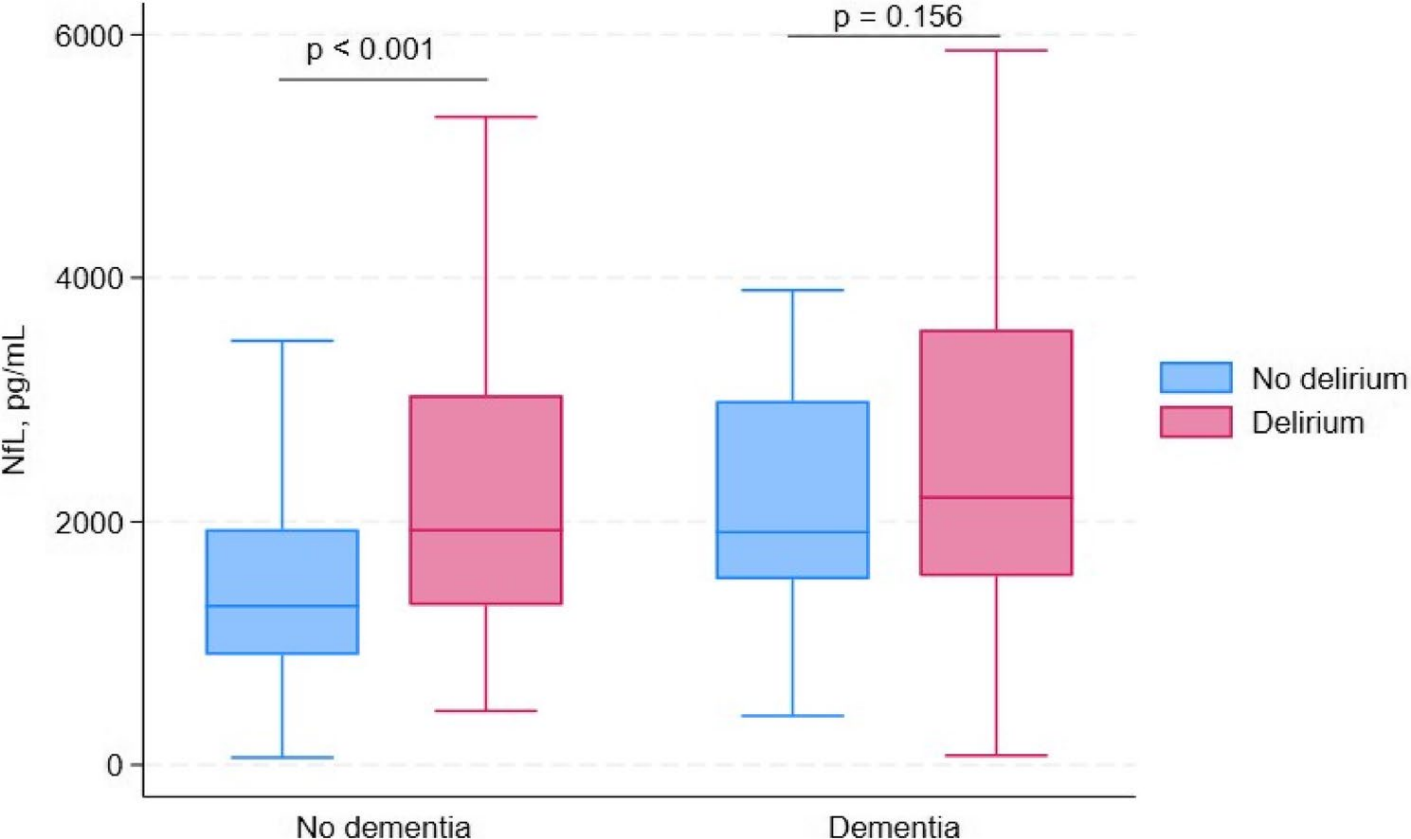
CSF-NfL concentrations in hip fracture patients with and without delirium stratified by dementia status (*N* = 496). Mann-Whitney U test for assessing differences in rank-order concentrations of NfL by delirium, stratified by dementia. The raw NfL data are shown in a Box-and-Whisker plot (outliers excluded) on the left side with the median (middle line) and interquartile range (box). The uppermost and lowermost lines represent the upper and lower adjacent values, calculated by adding or subtracting 1.5 times the interquartile range from the upper (75th percentile) and lower (25th percentile) quartile, respectively. This figure illustrates that NfL concentrations in delirium in hip fracture patients without dementia are similar to those in dementia without delirium, where dementia patients with delirium have only slightly higher concentrations than these groups

A *P*-value of < 0.05 was considered significant for all analyses. All analyses and Fig. 1 were produced using StataCorp. 2023. *Stata Statistical Software: Release 18*. College Station, TX: StataCorp LLC.

## Results

### Study participants

Demographics and clinical characteristics are described in Table 1. In total, 350 (70%) of the patients were women, and 221 (44%) had dementia. The mean age of the participants was 81 (standard deviation (SD) 10, age range: 41 to 101 years). The distribution by age category was as follows: 40–49 years (*n* = 4), 50–59 years (*n* = 10), 60–69 years (*n* = 53),70–79 years (*n* = 114), 80–89 years (*n* = 214), 90–99 years (*n* = 99), and ≥100 years (*n* = 2).

**Table 1:** Characteristics of patients with hip fracture according to delirium status (*N*=496^a^)

Variable	No delirium *N* = 237	Delirium *N* = 259	GC^b^	*P*-value
Demographics and clinical characteristics				
Age^c, d^ (years)	77.1^e^ ±10·9	84.8^f^ ±7.4	0.43	< 0.001**
Female ^g^	170 (72)	180 (69)	0.02	0.586
Dementia^g, h^, n (%)	30 (13)	191 (74)	0.61	< 0.001**
eGFR^c^ (ml/min/1.73 m2)	73.7 ± 20·8	66.8 ± 19.5	0.19	< 0.001**
ADL^i, j^	20 [1]	17 [6]	0.59	< 0.001**
ASA IV-III ^g^	70 (30)	158 (61)	0.34	< 0.001**
CSF NfL^i^ (pg/mL)	1366 [1029]	2116 [1848]	0.40	< 0.001**

Univariate associations between delirium and demographics and clinical characteristics were determined using the Student t-test (Age and eGFR), Pearson’s *X*^2^ (Female and IQCODE) and Mann-Whitney U test (ADL and CSF NfL)

*Abbreviations*: *ADL* Activities of daily living, *CSF NfL* Neurofilament light chain in the cerebrospinal fluid, *SD* Standard deviation, *eGFR* glomerular filtration rate, *GC* Gini Coefficient

^a^51 patients with subsyndromal delirium were excluded.

^b^ Gini coefficient ((2*Area under the curve)-1). The Gini coefficient is here comparable for all variables. The subsequent scale (0 to 1 or 0 to -1) represents effect sizes that can be considered weak (≥ 0.1 to < 0.3), moderate (≥ 0.3 to < 0.4), and strong (≥ 0.4)

^c^ Mean ±Standard deviation

^d^ Mean age for all the participants 81 years (SD 8), minimum 41 years, maximum 101 years. Distribution by age category: 40-49 years (*n*=4), 50-59 years (*n*=10), 60-69 years (*n*=53), 70-79 years (*n*=114), 80-89 years (*n*=214), 90-99 years (*n*=99), ≥100 years (*n*=2)

^e^ Minimum 41 years, maximum 100 years

^f^ Minimum 50 years, maximum 101 years

^g^ Number and (Percent) with trait according to delirium status

^h^Pre-fracture dementia status was assessed using the Informant Questionnaire on Cognitive Decline in the Elderly (IQCODE), with a cutoff of ≥ 3.44 indicating dementia. Missing data: IQCODE measured in 492 of 496 participants

^i^ Median and [interquartile range], i.e., the total distance between the p25 and p75 percentiles

^j^ Missing data: ADL measured in 462 of 496 participants

Statistically significant *P*-value < 0.05, ** *P*-value < 0.001

In total 496 patients were included in the main analysis (51 patients were excluded due to subsyndromal delirium). Of these, 259 (52%) were diagnosed with delirium. Delirium occurred in 70 (54%) of patients in the first cohort and in 189 (45%) of patients in the second cohort. Of all delirium cases, 132 (51%) had pre-operative delirium (prevalent delirium), and 115 (45%) developed delirium post-operatively (incident delirium). In 12 cases, incident or prevalent delirium status was indeterminate. Patients with delirium were on average 8 years older than patients without delirium (mean 85 versus 77 years; GC, a scaled version of the AUC from − 1 to 1, see Statistics) = 0.43, *P* < 0.001). The majority of the patients in the delirium group had dementia (74% versus 13%; GC = 0.61, *P* < 0.001) and had lower ADL scores than those in the non-delirium group (median 17 versus 20; GC = 0.59, *P* < 0.001). Furthermore, patients with delirium had lower eGFR (mean 67 versus 74 ml/min/1.73 m2; GC = 0.19, *P* < 0.001) compared with patients without delirium. Sex did not appear to influence delirium status (GC = 0.02, *P* = 0.586). During one year of follow-up, 122 out of 548 patients (22%) with hip fracture died.

### CSF neurofilament light chain concentrations in patients with delirium

CSF-NfL concentrations were significantly higher in the delirium group compared with the non-delirium group in univariate analysis (median 2116 pg/ml versus 1366 pg/ml; GC = 0.40, *P* < 0.001, Mann-Whitney U test (Table 1). However, when using multivariable logistic regression adjusted for age, sex, dementia, ADL, ASA, eGFR and cohort, the association between delirium and elevated CSF-NfL concentrations did not remain significant OR 1.29, CI [0.92, 1.81], *P* = 0.128) compared to univariate analysis (OR 2.21, CI [1.75, 2.78], *P* < 0.001, see Table 2). Notably, all clinical factors except sex, eGFR and cohort were highly significant, and their effect sizes were not attenuated much by the inclusion of CSF-NfL.

**Table 2:** CSF-NfL concentrations and clinical factors of delirium in patients with hip fracture (*N*=496^a^)

	Unadjusted model				Clinical predictors				Adjusted model		
	OR	CI	*P*		OR	CI	*P*		OR	CI	*P*
Age (years)					1.81	1.28, 2.55	0.001*		1.67	1.16, 2.40	0.005*
Female					0.66	0.36, 1.21	0.183		0.73	0.39, 1.34	0.316
eGFR (ml/min/1.73 m2)					1.08	0.80, 1.46	0.592		1.10	0.81, 1.49	0.513
ADL^b^					0.55	0.37, 0.82	0.003*		0.57	0.38, 0.84	0.005*
Dementia^c^					9.32	5.13, 16.93	< 0.001**		8.79	4.82, 16.01	< 0.001**
ASA III-IV					2.44	1.41, 4.19	0.001*		2.40	1.39, 4.15	0.002*
Cohort					0.99	0.53, 1.86	0.993		0.96	0.51,1.80	0.904
CSF NfL (pg/mL)	2.21	1.75, 2.78	< 0.001**						1.29	0.92, 1.81	0.128

*Abbreviations*: *ADL* Activities of daily living,* ASA* American Society of Anesthesiologists physical status classification,* CI* Confidence Interval, *CSF NfL* Neurofilament light chain in the cerebrospinal fluid, *eGFR *glomerular filtration rate, *OR* Odds Ratio, *P p* value

^a^ 51 patients with subsyndromal delirium were excluded

^b^ ADL was assessed by using the Barthel ADL Index

^c^ Pre-fracture dementia status was assessed using the Informant Questionnaire on Cognitive Decline in the Elderly (IQCODE), with a cutoff of ≥ 3.44 indicating dementia

Statistically significant *P*-value < 0.05*, ** *P*-value < 0.001

We further evaluated the association between CSF-NfL concentrations and delirium according to the timing of its clinical presentation (prevalent versus incident delirium). In the unadjusted analyses, higher CSF-NfL concentrations were associated with both prevalent and incident delirium (OR 2.27, CI [1.73, 2.99], *P* < 0.001 versus OR 2.03, CI [1.55, 2.65], *P* < 0.001, respectively). However, these associations were attenuated after adjusting for age, sex, dementia, ADL, ASA, eGFR, and cohort (OR 1.20, [0.80, 1.80], *P* = 0.361 versus OR 1.22, [0.82, 1.83] *P* < 0.313 respectively).

### CSF neurofilament light chain concentrations and one-year mortality

In the non-adjusted analysis, we found that CSF-NfL concentrations were associated with one-year mortality (Hazard Ratio (HR) 1.60 [1.37, 1.87] *P* < 0.001). These findings did not remain significant when adjusted for delirium, age, sex, dementia, ADL, ASA, eGFR, and cohort (HR 1.03, [0.84, 1.26], *P* = 0.736) where all clinical factors remained significant and were not attenuated much by the inclusion of CSF-NfL in the model (Table 3).

**Table 3:** CSF-NfL concentrations and clinical factors of mortality in patients with delirium (*N* = 548)

	Unadjusted model				Clinical predictors			Adjusted model		
	HR	CI	*P*		HR	CI	*P*	HR	CI	*p*
Age					1.32	0.98, 1.78	0.061	1.31	0.97, 1.77	0.076
Female					0.60	0.40, 0.91	0.016*	0.61	0.40, 0.92	0.019*
eGFR					0.77	0.62, 0.97	0.026*	0.78	0.62, 0.97	0.031*
ADL^a^					0.75	0.62, 0.90	0.003*	0.75	0.62, 0.91	0.003*
Delirium					2.12	1.19, 3.75	0.010*	2.10	1.18, 3.73	0.011*
Dementia^b^					2.05	1.19, 3.53	0.009*	2.03	1.18, 3.50	0.010*
ASA III-IV					1.30	0.83, 2.06	0.244	1.30	0.82, 2.05	0.252
Cohort					1.03	0.68, 1.58	0.862	1.03	0.67, 1.58	0.873
CSF NFL pg/mL)	1.60	1.37, 1.87	< 0.001**					1.03	0.84, 1.26	0.736

*Abbreviations*: *ADL* Activities of daily living, *ASA* American Society of Anesthesiologists physical status classification, *CI* Confidence Interval, *CSF NfL* Neurofilament light chain in the cerebrospinal fluid, *eGFR* glomerular filtration rate, *HR* Hazard Ratio, *CSF NfL* Neurofilament light chain in the cerebrospinal fluid, *P p* value

^a^ADL was assessed by using the Barthel ADL Index

^b^ Pre-fracture dementia status was assessed using the Informant Questionnaire on Cognitive Decline in the Elderly (IQCODE), with a cutoff of ≥ 3.44 indicating dementia

Statistically significant *P*-value < 0.05*, ** *P*-value < 0.001

### The impact of clinical factors

In evaluating the association between CSF-NfL, delirium (Table 2) and mortality (Table 3), CSF-NfL was highly significant. Assessing confounding from clinical factors, the percentage change in estimate in adjusted analyses one covariate at the time, age, ADL, ASA and dementia were important confounders for both the CSF-NfL-delirium and CSF-NfL-mortality associations, adjusted for cohort (Supplementary Table 3). Assessing all covariates, delirium was the strongest confounder of the association between CSF-NfL and mortality (16%). Multivariate analysis also showed age (20%) and dementia status (25%) as key confounders of the CSF-NfL-delirium association.

### CSF neurofilament light chain concentrations in patients with delirium according to dementia status

We performed analyses stratified by dementia status. In the univariate analysis, CSF-NfL concentrations were associated with delirium in patients without dementia (OR 2.14 [1.52, 2.99], *P* < 0.001). The findings remained significant in the multivariate analysis after adjustment for age, sex, ADL, eGFR, ASA and cohort (OR 1.84, [1.17, 2.89] *P* = 0.007). We did not find a CSF-NfL-delirium association either in the univariate (OR 1.10 [0.74, 1.65] *P* = 0.622) or the adjusted analysis (OR 0.83 [0.50, 1.39] *P* = 0.491) in the dementia group (Supplementary Table 1).

In the subgroup analysis, when comparing CSF-NfL concentrations in delirium stratified by dementia status, patients without dementia and without delirium had median CSF-NfL concentrations of 1267 pg/ml (IQR = 1042, *n* = 207). Concentrations of CSF-NfL were approximately similar in patients without dementia and with delirium and in patients with dementia without delirium (median 1930 pg/ml, IQR = 1722, *n* = 68 versus 1895 pg/ml, IQR = 1717, *n* = 191, respectively) (Fig. 1). Furthermore, the findings show that dementia patients with delirium have only slightly higher CSF-NfL concentrations than patients without dementia and with delirium (median 2199 pg/ml, IQR = 2022, *n* = 30 versus 1930 pg/ml, IQR = 1722, *n* = 68 respectively) (Fig. 1).

### CSF neurofilament light chain concentrations and one-year mortality according to dementia status

In the non-adjusted subgroup analysis comparing patients with and without dementia, CSF-NfL concentrations were associated with mortality in both groups (non-dementia group HR 1.46, [1.02, 2.09] *P* = 0.035; dementia group HR 1.37, [1.13, 1.67] *P* = 0.001). The findings did not remain significant after adjustment for age, sex, eGFR, ADL, ASA, delirium, and cohort (non-dementia group HR 0.89, [0.56, 1.25] *P* = 0.372; dementia group HR 1.10, [0.88, 1.39] *P* = 0.374) (Supplementary Table 2). One-year mortality rates were higher in patients with dementia (37%) than in patients without dementia (11%).

## Discussion

In this large multicenter study of patients with hip fracture, we examined the association between CSF-NfL concentration and delirium, and CSF-NfL one-year mortality in patients with hip fracture. Overall, CSF-NfL was not associated with delirium or one-year mortality, as most of the univariate associations could be explained by age, ADL and pre-existing dementia or delirium. However, in hip fracture patients without dementia, there was a significant association between CSF-NfL and delirium.

To our knowledge, the current study, along with the research by Halaas et al. [16] and Tsui et al. [17], are the only studies that have investigated CSF-NfL concentration in relation to delirium. In contrast to the findings of Halaas et al. [16] but in line with studies on blood NfL, CSF-NfL concentration was significantly higher in patients with delirium versus no delirium. The adjustment for confounders in previous studies has been variable. For instance, Brown et al. [59] studied delirium following cardiac surgery and did not adjust for pre-operative cognitive status, while Fong et al. [23] studied post-operative patients but solely performed a univariate analysis. Our study highlights the importance of the multivariable analysis, as NfL is also related to many of the factors that predispose clinically to delirium, such as age, dementia and ADL [1, 4, 14, 30, 32].

In line with the findings of the study by Fong et al. [23], we found a significant association between CSF-NfL and delirium, but only in patients without dementia. Several factors may account for these findings. Firstly, given that dementia is associated with elevated CSF-NfL concentrations [11, 13] and dementia is also recognized as a strong predisposing risk factor for delirium [8, 60, 61], it is likely that elevated CSF-NfL may be a marker of undetected dementia in the non-delirium group. Although dementia status was evaluated using the IQCODE, a validated assessment tool, it may not fully identify all cases of dementia, potentially leading to a degree of misclassification. The IQCODE with a cutoff of ≥ 3.44 has a sensitivity of 0.84 and a specificity of 0.80 [40], indicating that around 20% of participants in the control group may have undiagnosed dementia. Secondly, patients with advanced dementia and delirium may have higher CSF-NfL concentrations, but it may be difficult to detect delirium clinically, leading to a misdiagnosis of no delirium [62]. Thirdly, elevated baseline concentration of CSF-NfL related to chronic neurodegenerative disease may obscure transient changes in CSF-NfL in delirium. Despite the statistically significant association between CSF-NfL and delirium in non-demented patients, the predictive utility of CSF-NfL in this population appears to be weak. Therefore, CSF-NfL should likely not be considered as a biomarker of delirium although it is of interest to what degree it identifies patients with undetected neurodegeneration, a topic for future studies. Repeated measurements over time, possibly utilizing blood NfL, could help reveal differences in NfL concentration suggesting acute damage against a backdrop of chronically elevated NfL. However, if neurotoxicity associated with delirium were present, we would expect to observe considerably elevated NfL levels in patients who experienced delirium prior to surgery and thus exposed to delirium before the CSF measurement. This was not the case. Further, it will be of major interest to study whether elevated CSF-NfL during delirium is associated with later cognitive deficits. In terms of pathophysiology, elevated NfL concentrations reflect neuronal damage [11]. NfL is associated with delirium severity [19] and longer duration of delirium [21]. Our study cannot rule out neurotoxicity in delirium. However, the overall pattern of findings in our study is more consistent with elevated NfL being related to ageing and dementia among patients with delirium. Furthermore, in patients without dementia, somewhat elevated NfL concentrations are consistent with an expected number of sub-clinical and undiagnosed cases of neurodegenerative disease.

The univariate findings for CSF-NfL on mortality were no longer significant in the adjusted model, unlike findings in several other studies. For instance, Rübsamen et al. [28] found an association between CSF-NfL concentrations and all-cause mortality even after adjusting for covariates. The study by Page et al. [21], in which most participants had a positive CAM-ICU status, reported higher blood NfL concentrations in patients who died within six months after discharge. The authors concluded that NfL concentrations may help to identify patients with increased mortality risk. Similar to our study, several of these studies included patients with chronic neurodegenerative illness such as Parkinson or parkinsonian disorders [24, 27] and dementia [25, 26], and adjusted their analyses for age. Skillbäck et al. [25] and Bäckström et al. [27] also included sex in the adjusted models. Additionally, one study [28] investigated the NfL-mortality association in older individuals from the general population and included comorbidities and years of education along with age and sex in the multivariable model. The contrasting findings from our study regarding mortality and CSF-NfL concentrations may be attributed to several factors, including the length of follow-up period and the selection of covariates for the adjusted analyses, mainly dementia and delirium status. Previous studies linking CSF-NfL concentrations to mortality generally had a longer follow-up than one year 24–27.

The results from this study, both regarding the association between CSF-NfL concentrations and delirium, as well as between NfL and mortality, emphasize that age, dementia, and ADL are important confounding factors. Therefore, to appropriately interpret the link between CSF-NfL concentrations and delirium, it is crucial to assess how various factors that are related to delirium may influence CSF-NfL concentrations. By taking these confounding factors into account, we can improve the interpretation of CSF-NfL data in clinical and research contexts. However, confounding does not exclude a biological relevant role of neuronal injury as assessed by NfL in both delirium and mortality, as neuronal injury could clearly be negative for brain function even if it is related to underlying factors such as aging and neurodegenerative illness. Yet, such explanations imply causality, which cannot be established by observational studies.

Our study has several strengths, mainly the large sample size with CSF samples and a prospective evaluation of delirium using established instruments and diagnostic criteria that are effective in detecting delirium [63, 64]. Dementia status was assessed by using the IQCODE, a validated and frequently used assessment tool, and is considered to have a sensitivity of approximately 0.80 [40]. However, it is important to note that the IQCODE does not contain objective cognitive tests as part of the formal diagnostic process. This limitation may impact the evaluation of dementia status and could lead to some misdiagnosed dementia/no dementia cases. Residual confounding may also have been present in the group classified as not having dementia. Some patients in this group may nevertheless have had symptomatic cognitive impairment not detected by the IQCODE, or asymptomatic neurodegeneration for which NfL may serve as a biomarker. Hence, future studies with more robust cognitive and biomarker testing for neurodegeneration are needed to determine whether the observed association between NfL and delirium may, in part, reflect underlying neurodegenerative disease in this subgroup. In addition, as NfL may also be influenced by non neurological diseases [32], we adjusted for comorbidity using the ASA classification system as a proxy measure [46]. However, although the ASA score primarily reflects baseline systemic illness burden, it may also be influenced by acute perioperative factors.

Potential confounders related to biological, perioperative, and operation-related factors, such as preoperative medication use were not included in analyses. Systemic inflammatory biomarkers may also be related to NfL concentrations. Furthermore, other unmeasured potential confounding factors related to patients’ characteristics, such as diabetes, smoking, and body mass index (BMI), were also not included in our analyses. Evidence suggests that these variables may influence NfL levels [14, 30, 65–67]. However, the associations observed in our study were attenuated after adjusting for the confounders included in our study, already indicating significant confounding of the NfL delirium association. Future research should explore whether more extensive and complex analyses could explain more of this association. Indeed, surgery and anesthesia-related factors may also be particularly important to consider.

The classification of delirium subtype (hyperactive, hypoactive, or mixed) was available for only a limited subsample of participants. Consequently, we did evaluate whether variation in delirium subtype may have acted as a potential confounder influencing the findings of the study. CSF was sampled per operatively and, due to the ethical challenges of repeated sampling, it will be difficult to ascertain any temporal changes in CSF-NfL and the corresponding changes in, for example, delirium symptoms. Although performing repeated CSF measurements in large studies can be challenging, this approach may be conducted in small studies, as previously demonstrated by Fertleman et al. [68]. Unlike easily obtainable samples such as blood, CSF requires collection via lumbar puncture, an invasive procedure that makes it impractical for diagnosing delirium. However, a single measurement during acute illness could still offer valuable insights into the potential pathophysiological mechanisms involved in delirium. In addition, given the fluctuating nature of delirium, once-daily assessments may have been insufficient to capture all episodes, thereby resulting in some delirium episodes being missed.

In conclusion, the CSF-NfL delirium association is mostly explained by age and pre-existing cognitive impairment, with an association between CSF-NFL and delirium present only in patient without pre-existing dementia. However, this finding may be due to undiagnosed dementia cases, or subclinical neurodegeneration in this group. Indeed, having had delirium prior to CSF sampling was not related to NfL concentrations in CSF compared to developing delirium post-operatively. CSF-NfL was not independently associated with one-year mortality after hip fracture, with delirium being the main confounder of this association. This complex association suggests further research is needed to better understand the role of axonal injury in delirium. Our data suggests that elevated NfL concentrations in delirium can be mostly explained by factors such as age and cognitive impairment even as the association remained among patients without dementia.

## Supplementary Information

Supplementary Material 1.

## Data Availability

Due to ethical restrictions, the data set is available to the reader upon request only. Requests should be directed to the corresponding author at nathalbh@uio.no and data requestors will need to sign a data access agreement to gain access.

## Abbreviations

ADL: Activities of Daily Living
AUC: Area under the curve
CAM: Confusion Assessment Method
CSF: Cerebrospinal fluid
DSM-5: Diagnostic and Statistical Manual of Mental Disorders 5
eGFR: Glomerular filtration rate
GC: Gini coefficient
HR: Hazard ratio
IQCODE: Informant Questionnaire on Cognitive Decline in the Elderly
IQR: Interquartile range
NfL: Neurofilament light chain
OR: Odds ratio
REK: Regional Committee for Medical and Health Research Ethics
