# Supplementary material for: CSF neurofilament light chain concentration in patients with delirium following hip fracture: a multicenter prospective study
Source: BMC Geriatr. 2026 May 19;26:970. doi: 10.1186/s12877-026-07630-4 (PMC13390360; doi:10.1186/s12877-026-07630-4)
Supplement: Supplementary file 1 — Supplementary Material 1. [file 12877_2026_7630_MOESM1_ESM.docx]

**Supplementary material**

CSF neurofilament light chain concentration in patients with delirium following hip fracture; a multicenter prospective study

| **Supplementary Table 1. CSF-NfL concentrations in patients with hip fracture stratified by pre-fractur dementia status (N=496^a^)** | | | | | | | | |
| --- | --- | --- | --- | --- | --- | --- | --- | --- |
|  | | | | | | | | |
|  |  | **No dementia (n=275)^b^** | | |  | **Dementia (n=222)^b^** | | |
|  |  |  |  |  |  |  |  |  |
|  |  | **OR** | **CI** | **p** |  | **OR** | **CI** | **P** |
|  |  | | | | | | | |
|  | ***Unadjusted model*** | | | | | | | |
| CSF-NfL (pg/mL) |  | 2.13 | 1.52, 2.99 | <0.001** |  | 1.10 | 0.74, 1.65 | 0.622 |
|  |  | | | | | | | |
|  | ***Adjusted model*** | | | | | | | |
| Age (years) |  | 1.22 | 0.76, 1.94 | 0.398 |  | 2.50 | 1.34, 4.64 | 0.004* |
| Female |  | 0.81 | 0.38, 1.70 | 0.586 |  | 0.58 | 0.18, 1.83 | 0.361 |
| eGFR (ml/min/1.73 m2) |  | 0.94 | 0.63, 1.39 | 0.773 |  | 1.17 | 0.71, 1.91 | 0.525 |
| ADL^c^ |  | 0.58 | 0.34, 1.00 | 0.054 |  | 0.51 | 0.28, 0.91 | 0.024* |
| ASA IV-III |  | 2.58 | 1.28, 5.19 | 0.008* |  | 2.33 | 0.91, 5.95 | 0.076 |
| Cohort |  | 0.88 | 0.38, 2.04 | 0.779 |  | 1.25 | 0.46, 3.39 | 0.655 |
| CSF-NFL (pg/mL) |  | 1.84 | 1.17, 2.89 | 0.007* |  | 0.83 | 0.50, 1.39 | 0.491 |
| Abbreviations: ADL, Activities of daily living; ASA, American Society of Anesthesiologists physical status classification; CI, Confidence Interval; eGFR, glomerular filtration rate; CSF-NfL, Neurofilament light chain in the cerebrospinal fluid; OR, Odds Ratio; P, p value  ^a^51 patients with subsyndromal delirium were excluded.  ^b^ Pre-fracture dementia status was assessed using the Informant Questionnaire on Cognitive Decline in the Elderly (IQCODE), with a cutoff of ≥ 3·44 indicating dementia. Missing data: IQCODE measured in 492 of 496 participants  ^c^ ADL was assessed by using the Barthel ADL Index. Missing data: ADL measured in 462 of 496 participants.  Statistically significant *P*-value < 0·05, ** *P*-value < 0·001 | | | | | | | | |

| **Supplementary Table 2. CSF-NfL concentrations and mortality in patients with hip fracture stratified by pre-fractur dementia status (N=548)** | | | | | | | | |
| --- | --- | --- | --- | --- | --- | --- | --- | --- |
|  |  |  |  |  |  |  |  |  |
|  |  | **No dementia^a^** | | |  | **Dementia^a^** | | |
|  |  |  |  |  |  |  |  |  |
|  |  | **HR** | **CI** | **p** |  | **HR** | **CI** | **P** |
|  | ***Unadjusted model*** | | | | | | | |
| CSF-NfL (pg/mL) |  | 1.46 | 1.02, 2.09 | 0.035* |  | 1.37 | 1.13, 1.67 | 0.001* |
|  | ***Adjusted model*** | | | | | | | |
| Age (years) |  | 1.58 | 0.82, 3.04 | 0.167 |  | 1.23 | 0.85, 1.77 | 0.266 |
| Female |  | 0.18 | 0.07, 0.47 | <0.001* |  | 0.82 | 0.50, 1.33 | 0.426 |
| eGFR (ml/min/1.73 m2) |  | 0.63 | 0.40, 0.99 | 0.047* |  | 0.81 | 0.62, 1.06 | 0.137 |
| ADL^b^ |  | 1.58 | 0.84, 3.00 | 0.153 |  | 0.68 | 0.55, 0.84 | <0.001** |
| Delirium |  | 3.44 | 1.38, 8.53 | 0.008* |  | 1.88 | 0.88, 4.01 | 0.099 |
| ASA IV-III |  | 4.32 | 1.58, 11.83 | 0.004* |  | 0.89 | 0.53, 1.48 | 0.659 |
| Cohort |  | 0.57 | 0.21, 1.52 | 0.266 |  | 1.15 | 0.71, 1.86 | 0.547 |
| CSF-NFL (pg/mL) |  | 0.89 | 0.56, 1.25 | 0.372 |  | 1.10 | 0.88, 1.39 | 0.374 |
|  |  |  | | |  |  | | |
| Abbreviations: ADL, Activities of daily living; ASA, American Society of Anesthesiologists physical status classification; CI, Confidence Interval; CSF-NfL, Neurofilament light chain in the cerebrospinal fluid; eGFR, glomerular filtration rate; HR, Hazard Ratio; IQCODE, Informant Questionnaire on Cognitive Decline in the Elderly; CSF-NfL, Neurofilament light chain in the cerebrospinal fluid; P, p value  ^a^Pre-fracture dementia status was assessed using the IQCODE, with a cutoff of ≥ 3.44 indicating dementia.  ^b^ADL was assessed by using the Barthel ADL Index  ^b^ Pre-fracture dementia status was assessed using the IQCODE, with a cutoff of ≥ 3.44 indicating dementia.  Statistically significant *P*-value <0.05*, ** *P*-value <0.001 | | | | | | | | |

| **Supplementary Table 3. Confounding of CSF-NFL associations by known clinical factors (N=496^a^)** | | | | | |
| --- | --- | --- | --- | --- | --- |
|  |  | |  |  | |
|  | **Delirium** | |  | **One-year mortality** | |
| **Confounder** | **Univariate**  **CIE (%)** | **Multivariate**  **CIE (%)** |  | **Univariate**  **CIE (%)** | **Multivariate**  **CIE (%)** |
| Age (years)^b^ | 24 | 20 |  | 9 | 2 |
| Female ^b^ | 0 | 2 |  | 4 | 3 |
| eGFR (ml/min/1.73 m2) ^b^ | 5 | 0 |  | 8 | 7 |
| ADL^b,c^ | 25 | 5 |  | 10 | 2 |
| Dementia^b, d^ | 26 | 25 |  | 13 | 5 |
| ASA IV-III ^b^ | 14 | 2 |  | 11 | 6 |
| Delirium ^b^ |  |  |  | 16 | 16 |
| Abbreviations: ADL*,* Activities of daily living; ASA, American Society of Anesthesiologists physical status classification; CIE, Change in esitmate; CSF-NfL, Neurofilament light chain in the cerebrospinal fluid; eGFR, glomerular filtration rate; IQCODE, Informant Questionnaire on Cognitive Decline in the Elderly; CSF NfL, Neurofilament light chain in the cerebrospinal fluid.  ^a^ 51 patients with subsyndromal delirium were excluded.  ^b^ Adjusted for cohort.  ^c^ADL was assessed by using the Barthel ADL Index  ^d^ Pre-fracture dementia status was assessed using the IQCODE, with a cutoff of ≥3.44 indicating dementia. | | | | | |
